# Poly (ionic liquid)-Based Breath Figure Films: A New Kind of Honeycomb Porous Films with Great Extendable Capability

**DOI:** 10.1038/s41598-017-14563-x

**Published:** 2017-10-25

**Authors:** Baozhen Wu, Wanlin Zhang, Ning Gao, Meimei Zhou, Yun Liang, Ying Wang, Fengting Li, Guangtao Li

**Affiliations:** 1 College of Environmental Science & Engineering, Shanghai Key Laboratory of Chemical Assessment and Sustainability, College of Chemical Science and Engineering, Tongji University, Shanghai, 200092 China; 2 Department of Chemistry, Key Lab of Organic Optoelectronics & Molecular Engineering, Tsinghua University, Beijing, 100084 China; 3 Key Laboratory of Subsurface Hydrology and Ecological Effects in Arid Region, Ministry of Education, School of Environment Science and Engineering, Chang’an University, 710054 Xi’an, China

## Abstract

In this work, we reported a new method for the convenient fabrication of various functional porous films, which cannot be directly generated using breath figures (BFs). A series of polystyrene-b-poly (ionic liquid) (PS-b-PIL) block copolymers were employed for BFs process for the first time. It was found that PS-b-PIL could form well-defined BFs porous structure. Remarkably, the described PS-b-PIL copolymers are prone to form hierarchical structure, and the formed pore structure is strongly dependent on the used experimental parameters. Importantly, we found that the anion exchange could provide as an effective means, by which the porous films could be further and facilely converted into other functional films. As a demonstration, in our case, porous films with different surface (hydrophilic and hydrophobic) property, porous polydopamine films decorated with Au nanoparticles or glutathione and porous SiO_2_ films were prepared by using different counteranions as well as further conversion. Due to the unlimited combination of cation and anion in ionic liquid moiety, all the results indicate that the BFs films generated by using PS-PIL could serve as a platform to access various functional porous films by a simple counteranion exchange, showing a great extendable capability.

## Introduction

Breath figures (BFs) which is first reported in 1994 by François *et al*.^1^ which is a rapid, low-cost and versatile approach for efficiently producing regular porous honeycomb films. In the past two decades, honeycomb structured films have drawn considerable attention owing to their huge potential applications in a wide variety of fields, including as template^2^, separation^3^ and sensor^4^. Up to now, a widely recognized mechanism of BFs is described below. Firstly, the solution cooled and water vapor condensed, producing small and disordered water drops on the surface of the solution. Secondly, the water drops grew and self-assembled, forming a closely packed and regular array on the surface of the solution. Finally, the solvent and water drops evaporated successively, fabricating regular porous honeycomb film^5–7^. By using BFs approach, a large number of regular porous honeycomb films with various structures and morphologies have been fabricated^8–11^.

The materials suitable for the BFs process, however, are limited to soluble polymers and a small group of other materials^11^. A lot of functional materials cannot be directly utilized in the BF process. Thus, developing new strategy to access extended porous materials by exploiting the advantages of the BFs process is highly desirable. Few studies have been reported to address the relevant problems. For example, Li and co-workers use ferrocene and zinc acetylacetonate as precursors, Fe_2_O_3_ and ZnO inorganic porous honeycomb structures can be fabricated by pyrolysing corresponding PS-b-PAA/precursor composites breath figures arrays. These inorganic honeycomb patterns are used as either nuclear site or catalyst, which further direct the formation of CNTs patterns by chemical vapor deposition (CVD) and ZnO nanorod with a hydrothermal method^12–14^. Shimomura *et al*. developed a new kind of SERS substrates through simply peeling off the surface layer of the breath figures films followed by depositing silver on pincushion films. These periodically arranged structures of noble metals have proved to be good candidates as substrates for SERS^15^. Nevertheless, the strategy with great flexibility and expendability to access different BFs porous films, which cannot be directly generated using BFs process, is still limited.

**Figure 1:**
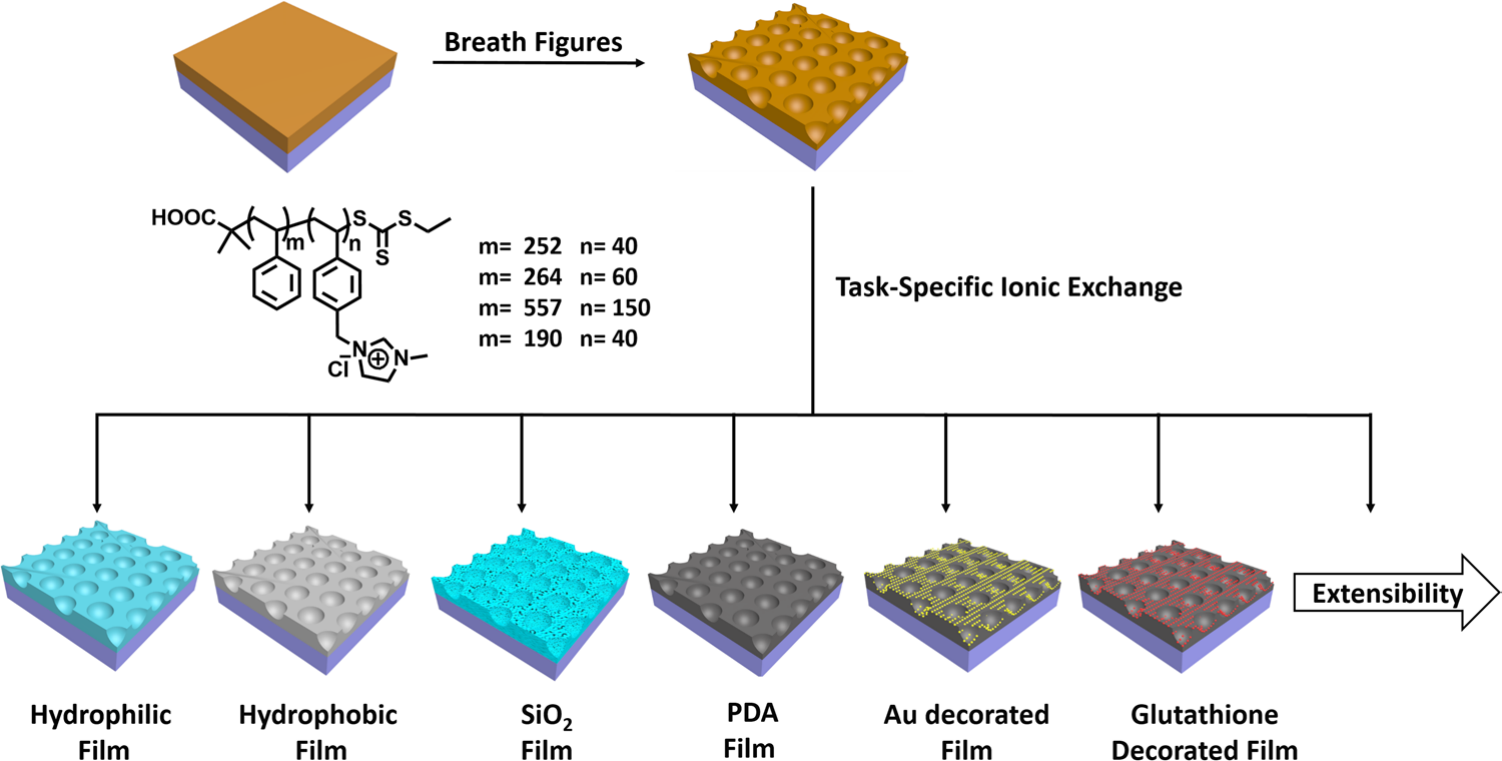
Schematic Representation of the preparation of BFs films by using PS-b-PIL and their functionalization.

In this work, based on a simple counteranion exchange reaction of ionic liquid moiety we developed a new approach for convenient fabrication of various functional porous films, particularly which cannot be directly generated using BFs process. Owing to their unique physical and chemical properties, polymerized ionic liquids (PILs) are emerging as a new type of materials showing great potential applications^16–18^. PILs combine the advantages of ionic liquid and polymers. One of the most distinct characteristics of PILs is that their properties can be facilely manipulated by a simple ion exchange reaction, providing a convenient and efficient means to access different polymeric materials. Additionally, block copolymer is an important type of amphiphilic molecules, which is suitable for BFs process and at the same time can form various nanostructures by self-assembly or micro-phase separation^17,18^. Thus, it is conceivable that the combination of both unique properties of IL and copolymer could enable the development of a new kind of BFs porous films, from which the fabrication of various other porous materials can be easily realized by simple ion-exchange or other followed reaction. To prove our concept, a series of polystyrene-b-poly (ionic liquid) (PS-b-PIL) block copolymers with various block ratios was synthesized and employed for BFs process for the first time (Fig. 1). Indeed, it was found that all of the synthesized amphiphilic PS-b-PIL block copolymers could stabilize water droplets during BFs process and produce well-defined BFs porous films. Remarkably, due to the salt feature of ionic liquid moiety and the resultant osmotic pressure, the described PS-b-PIL copolymers are prone to form hierarchical structure compared to the reported conventional polymers^11^, and the formed porous structure is strongly dependent on the used experimental parameters, including block ratio, solvent concentration, relative humidity, water pretreatment and post-treatment and salt solution pretreatment. The pore size and film morphology of honeycomb films could also be adjusted by post-treatment by swelling in hot water. Importantly, we found that the anion exchange could provide an effective means, by which the as-synthesized porous films from PS-b-PIL copolymers could be further and facilely converted into other functional films. As a demonstration, in our case, porous films with different surface (hydrophilic and hydrophobic) property, porous polydopamine films decorated with Au or glutathione and porous SiO_2_ films were prepared by using different counteranions as well as further conversion. All the results described here indicate that owing to the unlimited combination of cation and anion in ionic liquid moiety, the BFs films generated by using PS-b-PIL could serve as a versatile platform to access various functional porous films by a simple counteranion exchange, showing great extendable capability. Therefore, the proposed method here is very attractive for the preparation of functional and scalable films by the use of polystyrene-b-poly (ionic liquid) block copolymers and the combination of BFs and ion exchange.

## Results

### Fabrication of hierarchically structured polymer films

We synthesized a variety of polymerized ionic liquid block copolymers by completely ionization of polystyrene-b-poly (chloromethyl styrene) (PS-b-PCMS) diblock copolymers synthesized by RAFT according to previously reported procedures^20–24^.

**Figure 2:**
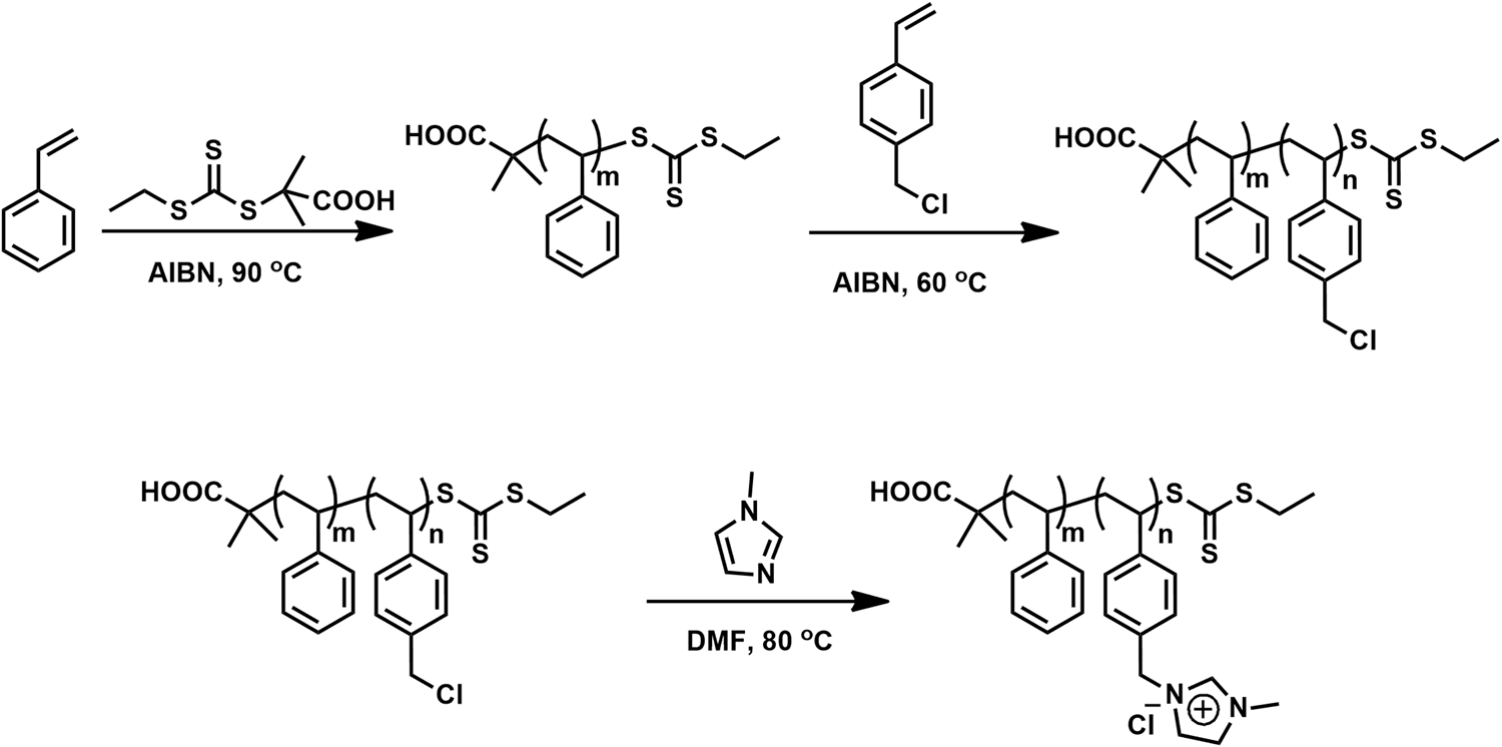
Synthesis of PS-b-PIL block copolymer by RAFT polymerization and ionization.

Four types of polymerized ionic liquid block copolymers named #1 PS252-b-PIL40, #2 PS264-b-PIL60, #3 PS557-b-PIL150 and #4 PS190-b-PIL95 were produced by RAFT and ionization (Fig. 2). Relative molecular weight and molecular weight distribution of PS-b-PCMS were determined by^1^H NMR and GPC. Relative molecular weight and molecular weight distribution of PS-b-PIL were calculated according the results described above after the ionization of PCMS, which are 35.0–93.0 kg/mol and 1.11–1.18, respectively (Table 1). These four types of PS-b-PIL with different ratios of PIL/PS (f_comb_ = 0.2, 0.3, 0.4 and 0.5) were used in the following breath figures process. It is generally accepted that the honeycomb porous structures are generated in following three steps during breath figures process^6,7^. Firstly, the solution coated on a substrate is cooled through solvent evaporation, and surrounding water vapor is condensed, producing small and disordered water drops on the surface of the solution. Secondly, the water droplets growth and self-assemble to from a closely packed and regular droplet array on the surface of the solution. Finally, the solvent and water drops evaporate successively, affording regular porous honeycomb film. The same mechanism should be suitable for the interpretation of the formation of the porous structures observed in our case.

**Table 1:** Four types of poly (ionic liquid) block copolymers synthesized by RAFT polymerization and ionization.

**Samples**	**PIL weight fraction f**_**comb**_^**a**^	**M**_**n**_ **(kg/mol)** ^**a**^	**PDI** ^**b**^
#1 PS252-b-PIL40	0.2	35.0	1.11
#2 PS264-b-PIL60	0.3	41.0	1.12
#3 PS557-b-PIL150	0.4	93.0	1.18
#4 PS190-b-PIL95	0.5	42.0	1.14

^a^Determined by quantitative ^1^HNMR. ^b^Determined by GPC using conventional calibration with PS standards.

### Morphological Characterization of honeycomb films

In this study, four types of PS-b-PIL with different PIL chain length were selected to prepare honeycomb porous membranes for the first time. PS-b-PIL block copolymers are quite unique since their distinct chemical structures are expected to have different performance during breath figures process, thus probably producing films with various interesting structures and morphologies. Due to the salt feature of ionic liquid moiety and the resultant osmotic pressure, the described PS-b-PIL copolymers are prone to form hierarchical structure which is different from the reported conventional polymers^11^ .The introduction of PS-b-PIL in breath figures can give a micro honeycomb structure and a nano-scale suborder on the wall of honeycomb films. The formed porous structure is strongly dependent on many experimental parameters.

**Figure 3:**
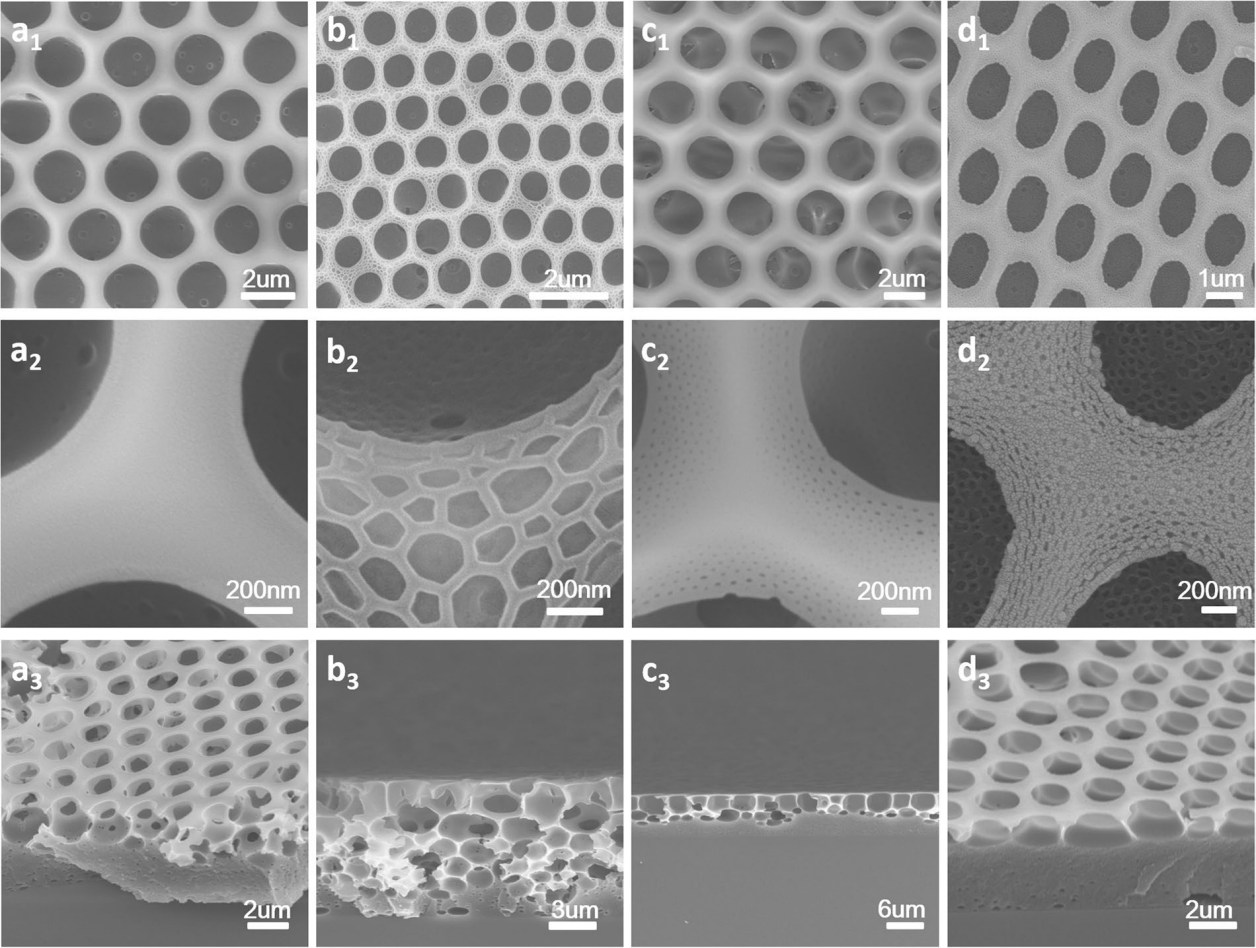
SEM images of the porous honeycomb films preparation by BFs from samples (**a**_**1**_) #1 PS252-b-PIL40; (**b**_**1**_) #2 PS264-b-PIL60; (**c**_**1**_) #3 PS557-b-PIL150 and (**d**_**1**_) #4 PS PS190-b-PIL95. (**a**_2_), (**b**_2_), (**c**_**2**_) and (**d**_**2**_) are corresponding magnified SEM images of (**a**_**1**_), (**b**_**1**_), (**c**_**1**_) and (**d**_**1**_). (**a**_**3**_), (**b**_**3**_), (**c**_**3**_) and (**d**_**3**_) are corresponding cross-section views of (**a**_**1**_), (**b**_**1**_), (**c**_**1**_) and (**d**_**1**_).

Breath figures process was generally performed in a chamber with steady temperature and relative humidity. During the breath figures process, solvent evaporated fast and water vapor condensed on the surface of the solution. The imprint of water drops on the solution and micelles of PS-b-PIL in the solution offered the films ordered honeycomb patterns and hierarchical structures. It is the first time to introduce imidazolium-functionalized poly (ionic liquid) block copolymer into breath figures. The obtained morphologies herein are quite different with that reported before^11^. The formation of a nano-scaled suborder was observed due to salt feature of ionic liquid moiety and the resultant osmotic pressure. The obtained honeycomb films with different ratios of PIL/PS showed different morphologies (Fig. 3) with the pore size of 0.8–2 μm in diameter.

**Figure 4:**
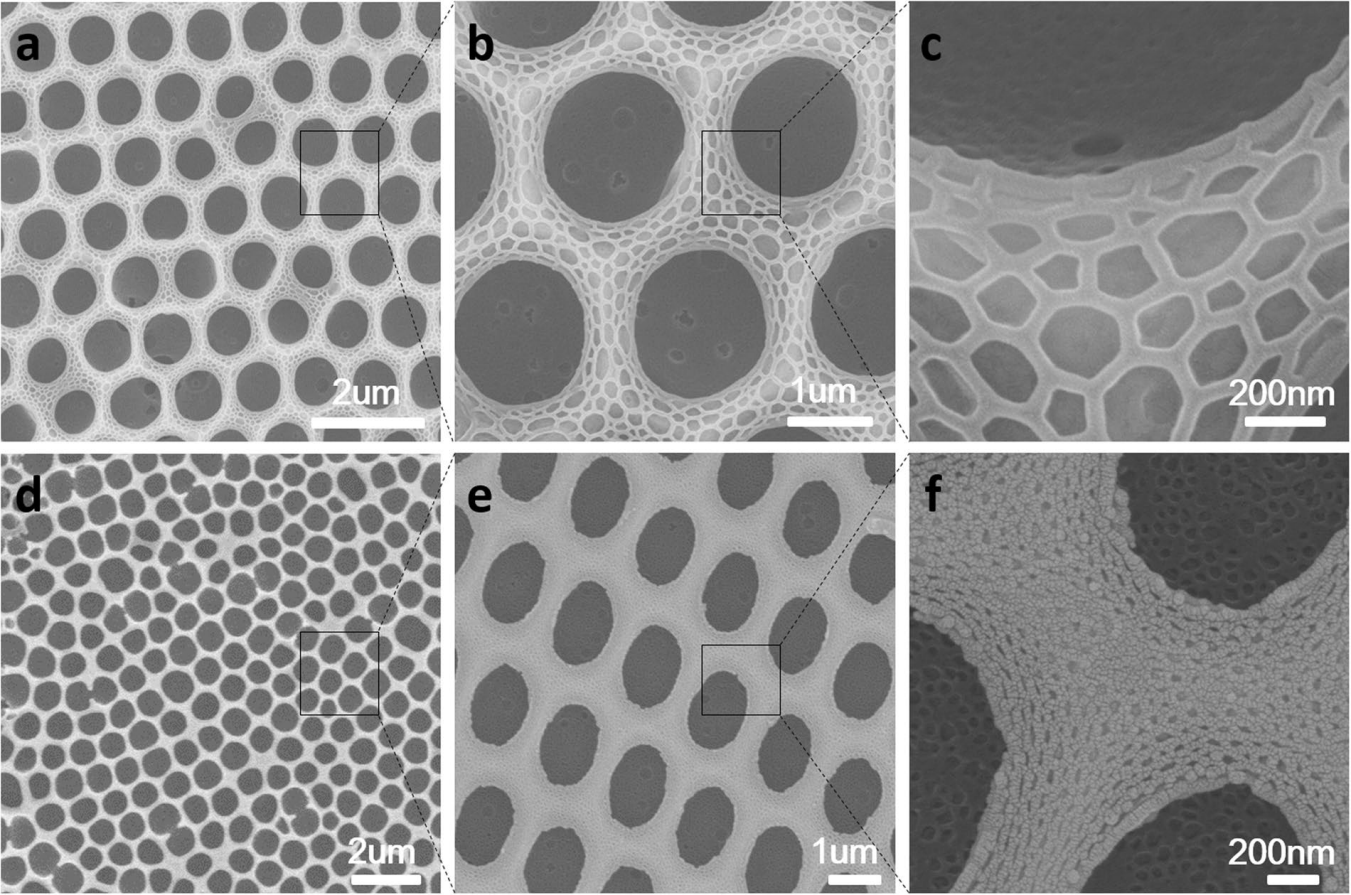
SEM images of the hierarchically structured polymer films prepared by BFs in different magnification from sample (**a**–**c**) #2 PS264-b-PIL60 and (**d**–**f**) sample #4 PS190-b-PIL95.

For the morphology of #1 PS252-b-PIL40, the pore distribution is regular with pore size of 2 μm and only slight nano structures in the pores were found. However, we can find obvious nano structures (50–100 nm) on the wall and inside the pore of honeycomb films of #2 PS264-b-PIL60 while the pore size decreased to 0.8 μm. From the cross section of #2, we can see layers of honeycomb films increased to 5 or 6 layers while for other three samples, only single layer were found. The pore sizes of #3 PS557-b-PIL150 and #4 PS190-b-PIL95 are about 2.4 um and 1 um, respectively. Nano structures on and inside the honeycomb films is about 20 nm. These structures are quite different from honeycomb films reported before^26^. We further compared the hierarchically structured honeycomb films generated from #2 PS264-b-PIL60 and #4 PS190-b-PIL95 (Fig. 4). The pore of #2 is more regular and nanostructures on and inside the honeycomb films is bigger than that of #4. The morphology and pore structure of PS-b-PIL honeycomb films were affected by many factors such as polymer concentration, block ratio, relative humidity, water saturation. The influence of these factors were systematically studied.

**Figure 5:**
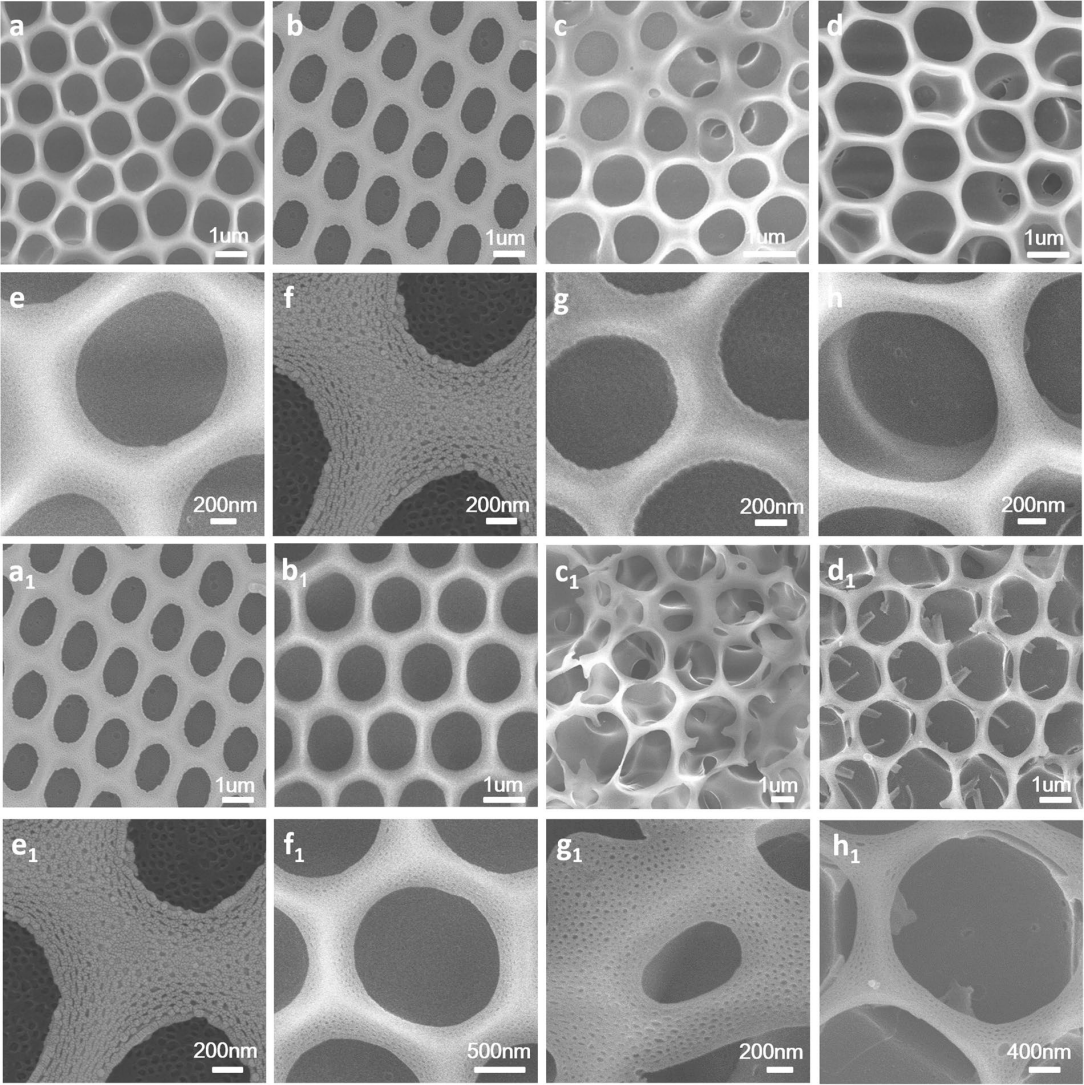
SEM images of honeycomb films fabricated by breath figures from sample #4 PS190-b-PIL95 in different concentrations and relative humidity. (**a**) 0.1 wt%; (**b**) 0.3 wt%; (**c**) 0.5 wt% and (**d**) 0.7 wt%. (**a**_**1**_) 35%; (**b**_**1**_) 45%; (**c**_**1**_) 55% and (**d**_**1**_) 65%. (**e**), (**f**), (**g**), (**h**), (**e**_**1**_), (**f**_**1**_), (**g**_**1**_), and (**h**_**1**_) are corresponding magnified SEM images of (**a**), (**b**), (**c**), (**d**), (**a**_**1**_), (**b**_**1**_), (**c**_**1**_) and (**d**_**1**_).

For the influence of polymer concentration on the pore structure and morphology of the porous BFs films, a series of #4 PS190-b-PIL95 solutions with different concentrations (0.1 wt%, 0.3 wt%, 0.5 wt% and 0.7 wt%) were used to fabricate films (Fig. 5a–h). When the concentration is 0.3 wt%, we can get regular honeycomb film with rich and regular nanostructures. When the solution concentration is lower to 0.1 wt% or higher to 0.5 and 0.7 wt%, the honeycomb films are irregular. These phenomena can be explained by the interaction between water vapor and polymer. Low concentration of polymer in the solution cannot provide sufficient number amphiphilic molecules for the stabilization of water droplets while high concentration of polymer renders the lack of solvent to evaporate for reducing the temperature, leading to that there is not enough water droplet to condense and the water droplet cannot arrange well on the solution^27^.

Relative humidity that could be adjusted by humidity detector in the chamber also has a big impact on the morphology of the honeycomb film. The mutual effect between the PIL and the relative humidity was responsible for the subtle equilibrium during the BFs process, which led to regular pores at low humidity (35–45%) and prevented high pores regularity at high humidity (above 55%), as depicted in Fig. 5a1–h1. Honeycomb films generated using #4 PS190-b-PIL95 at humidity of 35% show regular pores of 1 μm. As the humidity increases to 45%, more regular pores of 1 μm were generated. When we further increased the relative humidity to 55% and 65%, the pores were extensively large and their size were no longer uniform, totally blemishing the honeycomb porous arrangement. During breath figures process, the pores generated in honeycomb films were due to water droplets template, and these water droplets came from the surrounding humid environment^7^. Hence, the relative humidity has a big influence on the morphology of the honeycomb film. At relative lower humidity (35–45%), these water droplets can increase in size before polymer precipitation into solid layers. The polymers around the droplets can stabilize the water droplets very well. As a result, regular porous structure in polymer film is formed. However, when the relative humidity was too high (for example above 55%), the water droplets condensed on the surface may become surplus. In such case, a large number of the formed droplets could easily and rapidly coalesce before the occurrence of the precipitation of the polymer around the droplets, thus leading to the formation of irregular and larger pore structure. In agreement with the observation in our work, the similar phenomenon was also found in literature^26^.

**Figure 6:**
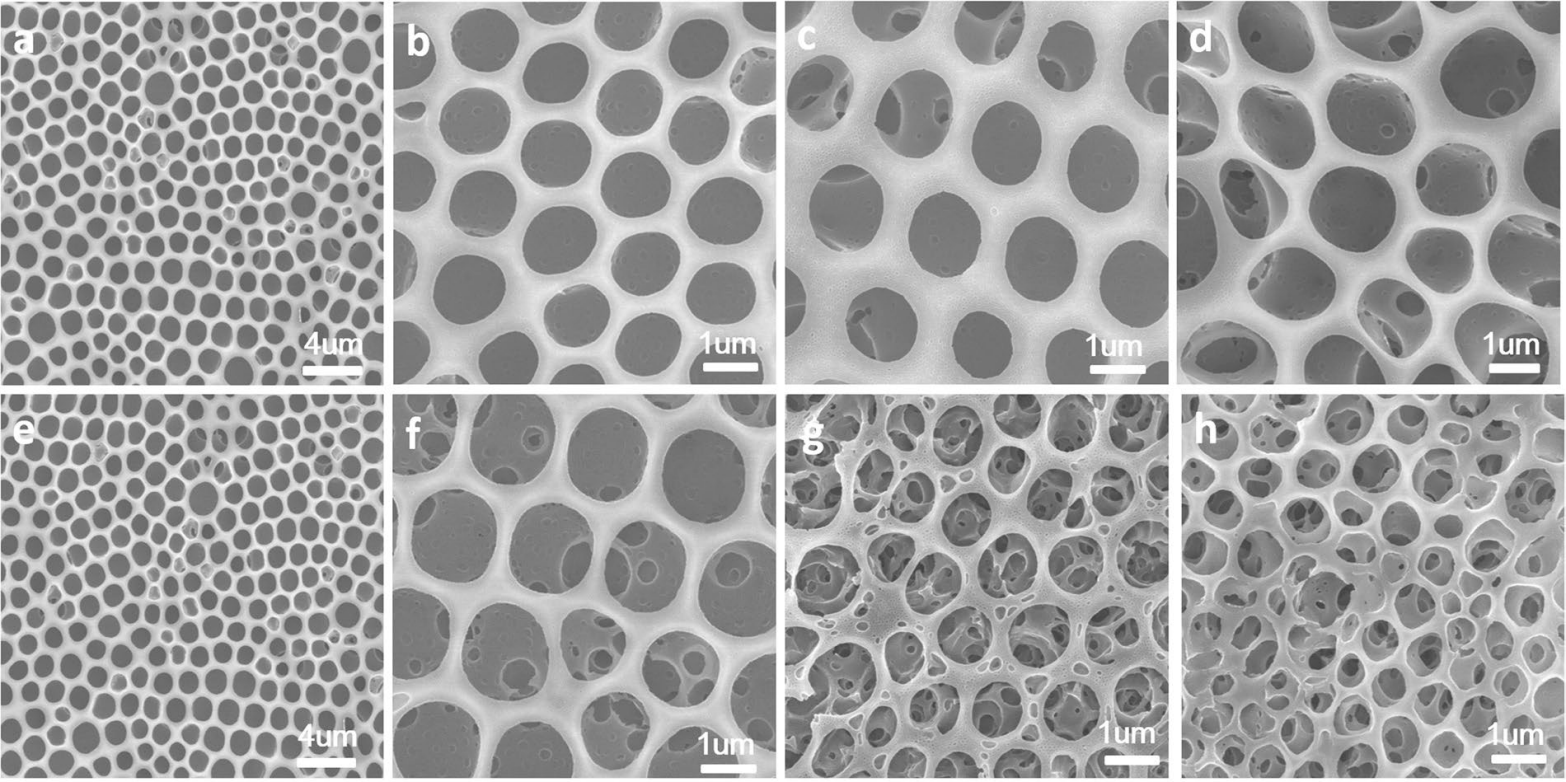
SEM images of porous honeycomb films prepared by BFs from sample #4 PS190-b-PIL95 with different water content and osmotic pressure. (**a**–**d**) with different water saturation (**a**) 0 μL; (**b**) 3 μL; (**c**) 6 μL; (**d**) 9 μL; (**e**–**h**) with different osmotic pressure (**e**) 0 mg; (**f**) 10 mg; (**g**) 20 mg; (**h**) 30 mg.

Since the honeycomb films were fabricated by PS-b-PIL, which owned the properties of ionic liquids, the morphology of the film was also considerably influenced by water content and osmotic pressure, which could be adjusted by adding different amounts of deionized water and saturated sodium chloride solution. During the process of breath figures of PS-b-PIL, different water contents in the solution and the differences of osmotic pressure between the inner and outer water phase have played an important role during the formation of honeycomb films. From Fig. 6a–d, various honeycomb films were obtained from precursor solution by adding 0 μL, 3 μL, 6 μL and 9 μL water. We can obviously see that water content has an important impact on the structure and morphology of honeycomb film. When we adding 3 μL and 6 μL deionized water, the films are more regular, and the pore size is about 1.2 μm. However, when we adding 9 μL deionized water, film morphology is irregular and the pore sizes are non-uniform. These result proved the water content has a big influence on honeycomb films fabricated by breath figures. Besides water content, we also studied the influence of osmotic pressure by adding different amount of saturated sodium chloride solution. From Fig. 6e–f, honeycomb films were obtained from precursor solution with adding 0 mg, 3 mg, 6 mg and 9 mg sodium chloride solution. We find that solutions without adding saturated sodium chloride solution yield breathe figures structures with single layer honeycomb films, as shown in Fig. 6e. On the contrary, PS-b-PIL solutions containing salt yield a multi-layer film with obvious hierarchical pore structure on the wall of honeycomb films (Fig. 6f–h). We tried to figure out why adjusting the above two effects can exhibit such morphologies of the films. Chloroform solution of polymer is usually employed for breath figures process. In our case, the used polymer is a poly(ionic liquid) containing block copolymer (PS-b-PIL), which shows distinct amphiphilic feature and can be used as a polymeric surfactant to form stable W/O emulsions. Thus, in our work, besides the chloroform solution of PS-b-PIL, the PS-b-PIL stabilized emulsions of water-chloroform and saturated NaCl solution-chloroform were also used for breath figures process with the expectation of creating new pore structure. Interestingly, we found that the water content in the used water-chloroform emulsions has an important influence on the formation of the pore structure in the resulting PS-b-PIL polymer films. When the water content is relatively small, the pore structure formed is generally irregular. Upon the increase of the amount of water, the pore structure became uniform at first, and then the further increase of water amount resulted in the destruction of the water droplets template arrangement, affording relative large and irregular pore structure. In the case of saturated NaCl-chloroform emulsion, it was found that the amount of saturated sodium chloride solution in the used emulsion has significant influence on the formation of the pore structure in the resulting PS-b-PIL polymer films. Different from the BFs process of using normal PS-b-PIL chloroform solution, probably due to the presence of osmotic pressure between the inner NaCl water phase and outer condensed water, the addition of saturated NaCl solution in PS-b-PIL chloroform could lead to dramatic increase of the pores in the formed polymer films. Especially, when increased amount of saturated NaCl solution was used, the polymer films with interconnected pore structure were obtained. The exact mechanism underlying for the evolution of pore structure is still not clear, and further more experiments are required.

**Figure 7:**
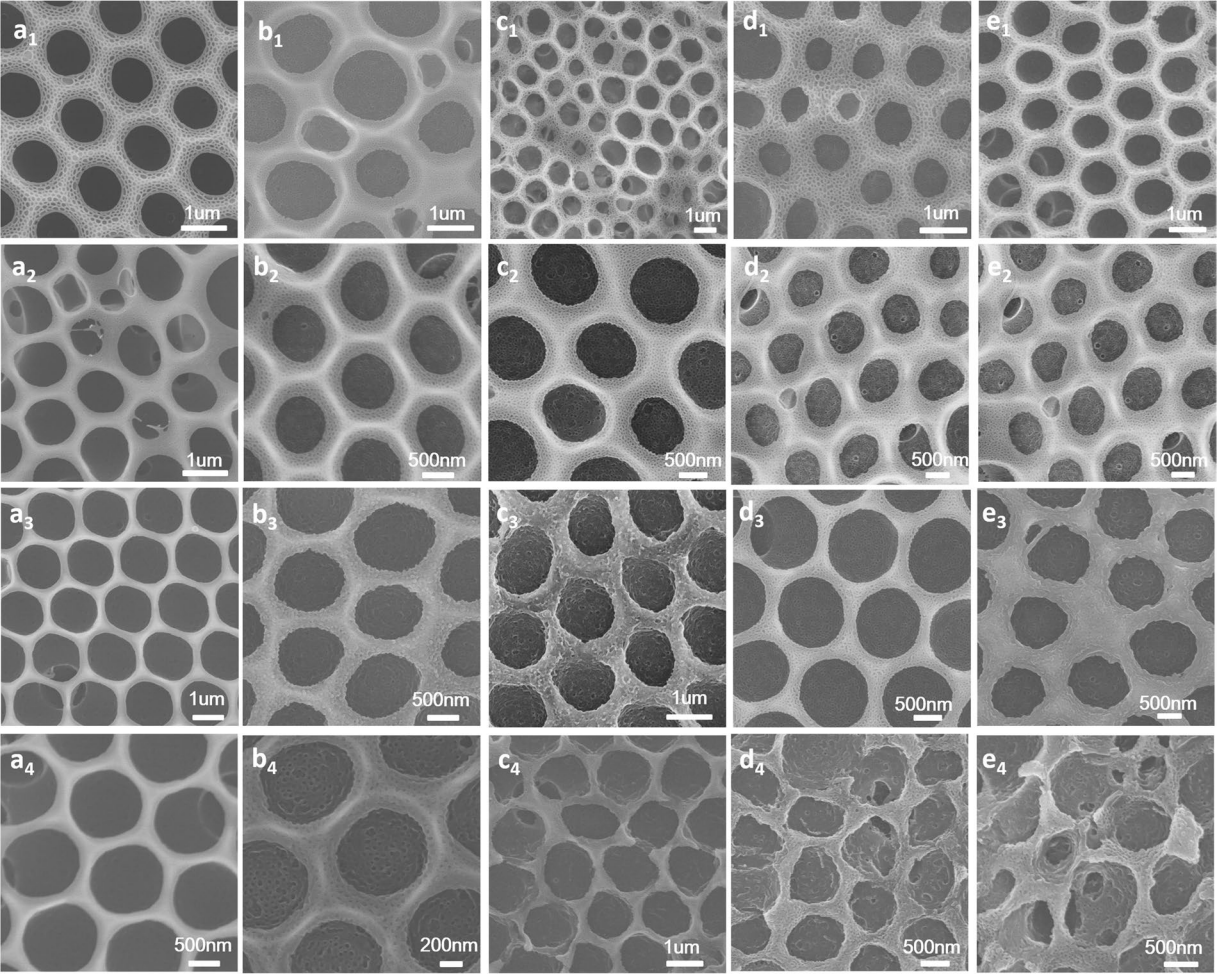
SEM images of porous honeycomb films prepared by BFs from sample #2 PS264-b-PIL60 without or with water saturation and in different swelling time in hot water. (**a**_**1**_–**e**_**1**_) without water saturation after swelling in hot water for (**a**_**1**_) 0 h; (**b**_**1**_) 1 h; (**c**_**1**_) 2 h; (**d**_**1**_) 3 h; (**e**_**1**_) 4 h. (**a**_**2**_–**e**_**2**_) with water saturation after swelling in hot water for (**a**_**2**_) 0 h; (**b**_**2**_) 1 h; (**c**_**2**_) 2 h; (**d**_**2**_) 3 h; (**e**_**2**_) 4 h. SEM images of porous honeycomb films prepared from sample #4 PS190-b-PIL95. (**a**_**1**_ **–e**_**1**_) without water saturation after swelling in hot water for (**a**_**3**_) 0 h; (**b**_**3**_) 1 h; (**c**_**3**_) 2 h; (**d**_**3**_) 3 h; (**e**_**3**_) 4 h. (**a**_**4**_–**e**_**4**_) with water saturation after swelling in hot water for (**a**_**4**_) 0 h; (**b**_**4**_) 1 h; (**c**_**4**_) 2 h; (**d**_**4**_) 3 h; (**e**_**4**_) 4 h.

**Figure 8:**
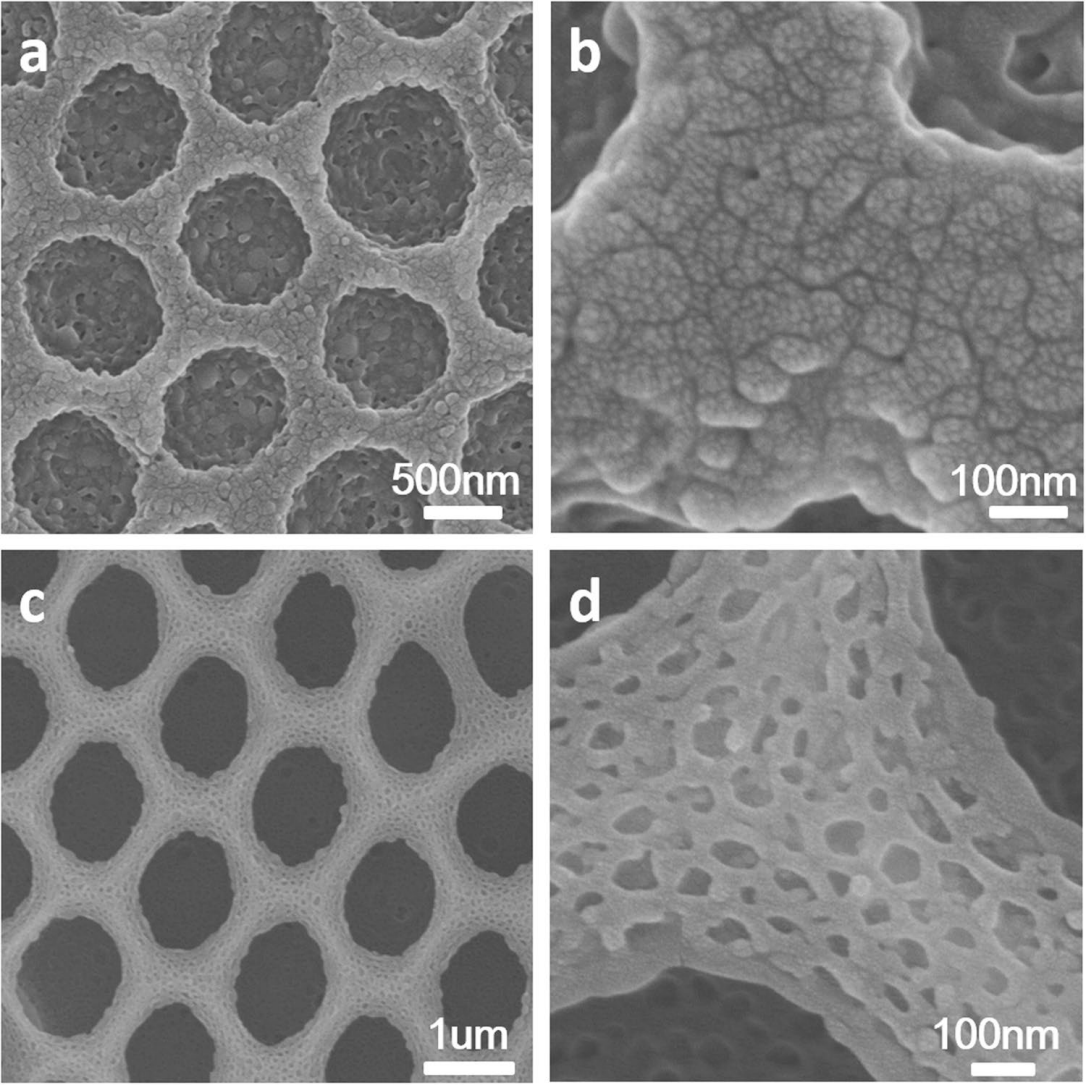
SEM images of the hierarchically honeycomb films prepared by BFs and swelling in hot water for 5 hours in different magnification from (**a**) #2 PS264-b-PIL60; and for 3 hours from (**c**) #4 PS190-b-PIL95. (**b**) and (**d**) are magnified SEM images of (**a**) and (**c**).

Poly (ionic liquid) block copolymer owned the properties of block copolymers and thus the morphology of the film was also considerably affected by micro-phase separation of block copolymer. Hot water was chosen as solvent to swell the honeycomb films because of the different interactions of blocks with hot water. After the process of breath figures, the honeycomb films of PS-b-PIL were put into hot water at 90 °C for different times. For further study the effects of block ratio, water pretreatment and post-treatment on the films, we selected films with and without water pretreatment to carry out the next treatment. From Fig. 7, porous honeycomb films prepared by BFs from sample #2 PS264-b-PIL60 and #4 PS190-b-PIL95 were pretreated without or with water saturation and swelled in hot water for different time. Honeycomb films of #2 PS264-b-PIL60 were pretreated without and with water saturation after swelling in hot water for 0 to 4 h (Fig. 7a1–e2). The films without water saturation showed a big change on nanostructure than films with water saturation. When films swelled for 3 and 4 hours, their nanostructure became soft. As time increased to 5 hours, the nanostructure disappeared and only cracked texture was found on the wall of honeycomb films (Fig. 8a,b). Honeycomb films of #4 PS190-b-PIL95, which is pretreated without and with water saturation after swelling in hot water for 0 to 4 h (Fig. 7a3–e3). The films without and with water saturation both showed big changes on nanostructure. When swelling time is 4 hours for films without water pretreatment and 3 or 4 hours for films with water pretreatment, the honeycomb structure also cracked. With swelling time for 3 hours, the nanostructure cracked. We can see the details in Fig. 8c,d. These results can be understood that the different length of the poly (ionic liquid) segments led to different interaction when swelling in hot water. A more thorough mechanism will continue to be studied in our future work.

The generation of membrane containing desired pore and pore distribution by BFs is a multi-factor process. Many parameters can adjust the pore size, such as different chain length, polymer concentrations, relative humidity water pre or post treatment and salt pretreatment and osmotic pressure.

### Fabrication of polymer films with different surface property

**Figure 9:**
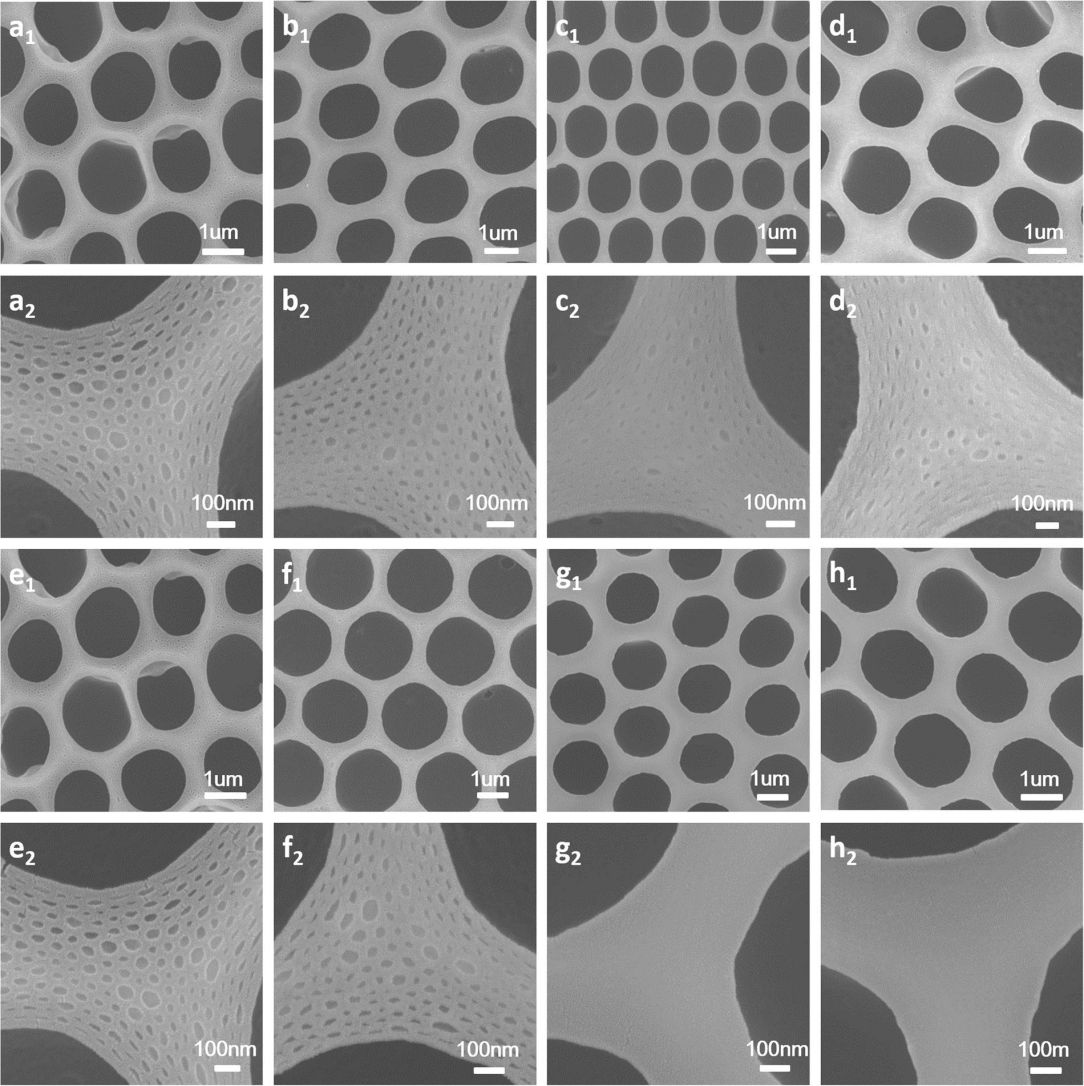
SEM images of honeycomb films after ionic exchange at different time fabricated from sample #4 PS190-b-PIL95 (**a**_**1**_–**d**_1_) 24 h; (**e**_**1**_–**g**_**1**_) 48 h. (**a**_**1**_) and (**e**_**1**_) without ionic exchange. (**b**_**2**_) and (**f**_**2**_) OH^−^ ionic exchange; (**c**_**1**_) and (**g**_**1**_) BF_4_^−^ ionic exchange; (**d**_**1**_) and (**h**_**1**_) Tf_2_N^−^ ionic exchange; (**a**_**2**_–**h**_**2**_) are magnified SEM images of (**a**_**1**_–**h**_**1**_).

**Figure 10:**
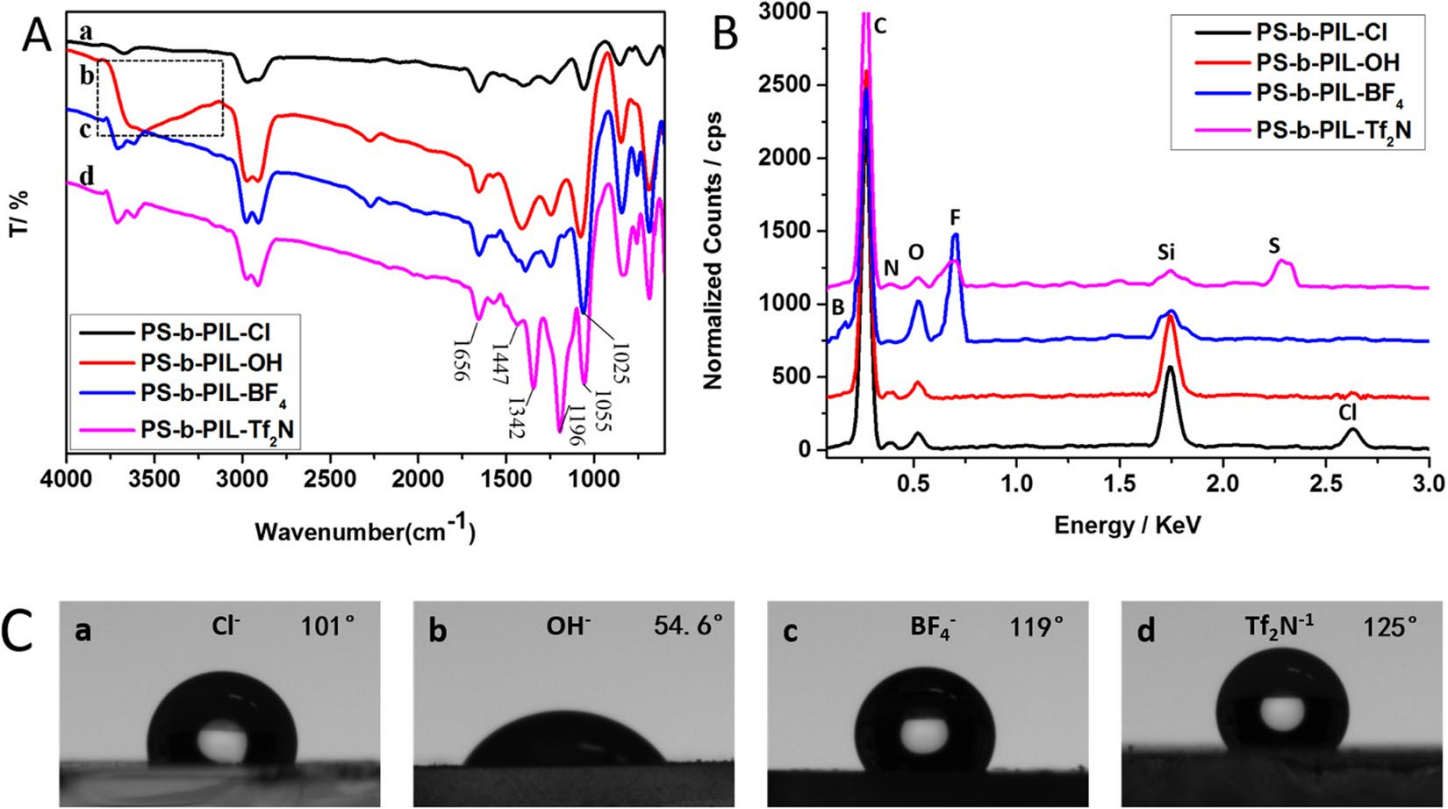
(**A**) ATR-FTIR spectrum of (a) PS-b-PIL-Cl, (b) PS-b-PIL-OH, (c) PS-b-PIL-BF_4_ and (d) PS-b-PIL-Tf_2_N after anion exchange. (**B**) Energy dispersive X-ray (EDX) spectra for polymerized ionic liquid membrane with different anion (a) PS-b-PIL-Cl, (b) PS-b-PIL-OH, (c) PS-b-PIL-BF_4_ and (d) PS-b-PIL-Tf_2_N with glass as the substrate. (**C**) Contact angle of polymerized ionic liquid membrane with different anion (a) PS-b-PIL-Cl, (b) PS-b-PIL-OH, (c) PS-b-PIL-BF_4_ and (d) PS-b-PIL-Tf_2_N with glass as the substrate.

The honeycomb films with different surface (hydrophilic and hydrophobic) property were obtained via counter anion exchange from the Cl^−^ to OH^−^, BF_4_^−^ and Tf_2_N^−^. The produced membranes are denoted as PS-b-PIL-OH, PS-b-PIL-BF_4_ and PS-b-PIL-Tf_2_N. The SEM images of PILs before and after counter anion exchanges were shown in Fig. 9. After the complete exchange, honeycomb films of PS-b-PIL-OH changed little and the nanostructure kept well on the film. However, for honeycomb films of PS-b-PIL-BF_4_ and PS-b-PIL-Tf_2_N, nanostructure on their films disappeared. After ionic exchange reaction for 24 hours, the ion may be not exchanged completely. While for 48 hours, the ionic exchange reaction is obviously completed, ATR-FTIR and EDS can further prove this (Fig. 10A,B). All the membranes show the absorption peaks at 1447 cm^−1^ and 1656 cm^−1^, which are the vibrational mode of the benzene ring and imidazolium cations. Line b in Fig. 10A shows a wide peak between 3700–3150 cm^−1^, which is due to the stretching vibration of O-H groups, indicating the anion exchange from Cl^−^ form to OH^−^ form. Energy-dispersive X-ray (EDX) spectra was used here to auxiliary prove the anion exchange reaction. The results show that no Cl and K elements in the membranes of OH^−^ form, which further confirms the thorough anion exchange in the films. The wide peak at 1025 cm^−1^ is the vibrations of BF_4_^−^, which indicate the anion exchange from Cl^−^ form to BF_4_^−^ form. No Cl and Na elements were detected in the BF_4_^−^ form film. At the same time, the appearance of new bands peaks at 1055 cm^−1^, 1196 cm^−1^ and 1342 cm^−1^ can be attributed to Tf_2_N^−^ anions. From EDX spectra in Fig. 10B, all the polymerized ionic liquids contained elements of corresponding anions, which further confirms the successful anion exchange of the PS-b-PIL block copolymers^29–34^. The hydrophilic and hydrophobic property of the films can be determined by contact angle (Fig. 10C). After anion exchange from Cl^−^ to OH^−^, the contact angle changed from 101° to 55° and the hydrophilic property greatly increased. When the counteranions were BF_4_^−^ and Tf_2_N^−^, the honeycomb films were more hydrophobic.

### Fabrication and application of functional honeycomb structured films

**Figure 11:**
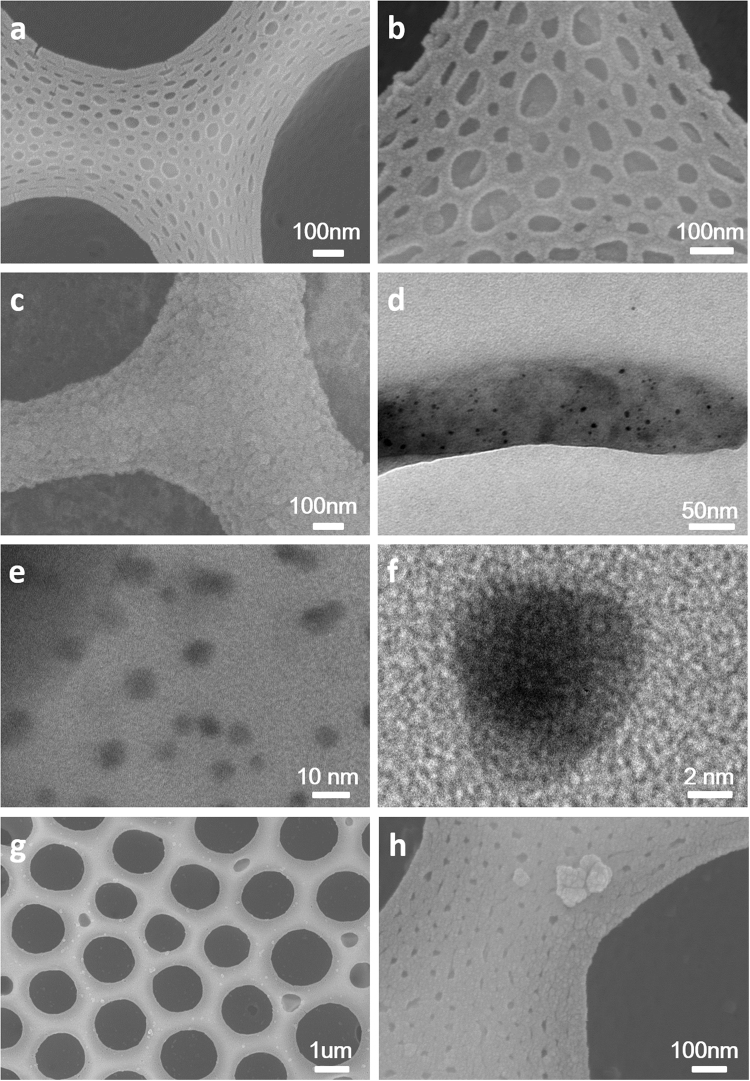
SEM images of the hierarchically polymer membranes prepared from sample #4 PS190-b-PIL95 after functionalization. (**a**) PS-b-PIL-OH; (**b**) PS-b-PIL-OH@PDA; (**c**) PS-b-PIL-OH@PDA@Au. (**d**) TEM images of PS-b-PIL-OH@PDA@Au; (**e**) High-resolution TEM images of PS-b-PIL-OH@PDA@Au; (**f**) the magnified high-resolution TEM images of (**e**); (**g**) PS-b-PIL-OH@PDA@GSH; (**h**) the magnified SEM images of (**g**).

We extend the functionalization of honeycomb films by preparation of PS-b-PIL-OH@PDA@Au. The hierarchically structure of PS-b-PIL-OH@PDA allowed well dispersions of Au nanoparticles and other catalytic nanoparticles for many potential applications. SEM, TEM and high-resolution TEM images of PS-b-PIL-OH@PDA@Au are presented in Fig. 11a–f. The changing of morphology of honeycomb films confirm the above findings (Fig. 11b,c). From the TEM images, we can see that the size of gold nanoparticles is about 5 nm ± 0.8 nm with no obvious aggregation.

**Figure 12:**
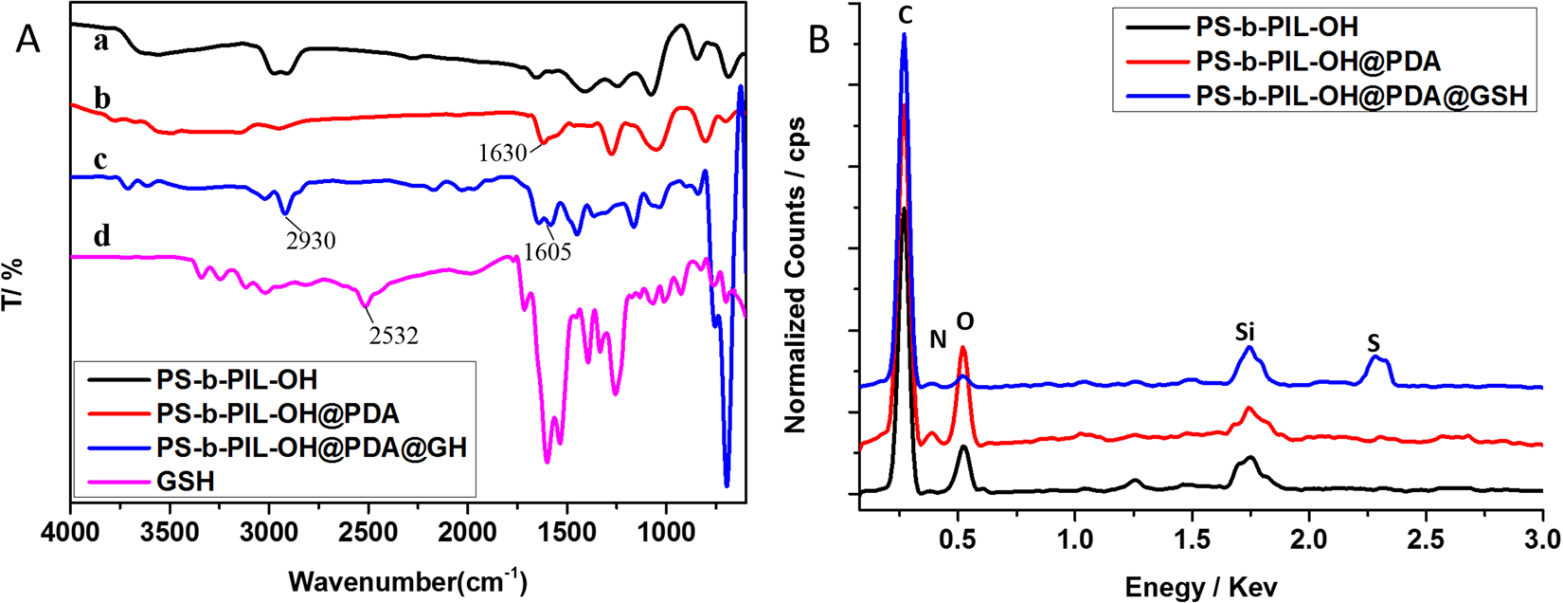
(**A**) ATR-FTIR spectrum of (a) PS-b-PIL-OH, (b) PS-b-PIL-OH@PDA, (c) PS-b-PIL-OH@PDA@GSH and (d) GSH after functionalization. (**B**) EDX spectra for (a) PS-b-PIL-OH, (b) PS-b-PIL-OH@PDA and (c) PS-b-PIL-OH@PDA@GSH.

The coating of GSH altered little to the pore structure (Fig. 11g,h). The introduction of GSH on the film was confirmed by ATR-FITR and EDX analysis of PS-b-PIL-OH, PS-b-PIL-OH@PDA, PS-b-PIL-OH@PDA@GSH and GSH membranes (Fig. 12). The formation of PDA on PS-b-PIL-OH can be corroborated by the appearance of absorption peak at 1630 cm^−1^, attributed to the stretching vibration of the C=C indole group, which is generated after polymerization reaction (Fig. 12A). In the 3500 cm^−1^ region, a broad absorption peak belonged to the O-H groups. Introduction of glutathione (GSH) on the films was also confirmed by ATR-FTIR spectroscopy. The characteristic peaks of 2930 cm^−1^ (C-H stretching), 1648 cm^−1^ (cysteine-carbonyl), and 1605 cm^−1^ (glutamic acid-carbonyl) proved the coating of GSH on PS-b-PIL-OH@PDA films. Thiol groups tightly anchored GSH. The ATR-FTIR peak at 2532 cm^−1^ for S-H stretching disappearing. EDX in Fig. 12B can be seen that all the functionalized of polymerized ionic liquids films are composed of desired characteristic element, which further confirms the successful modification GSH on PS-b-PIL-OH@PDA films^35,36^.

**Figure 13:**
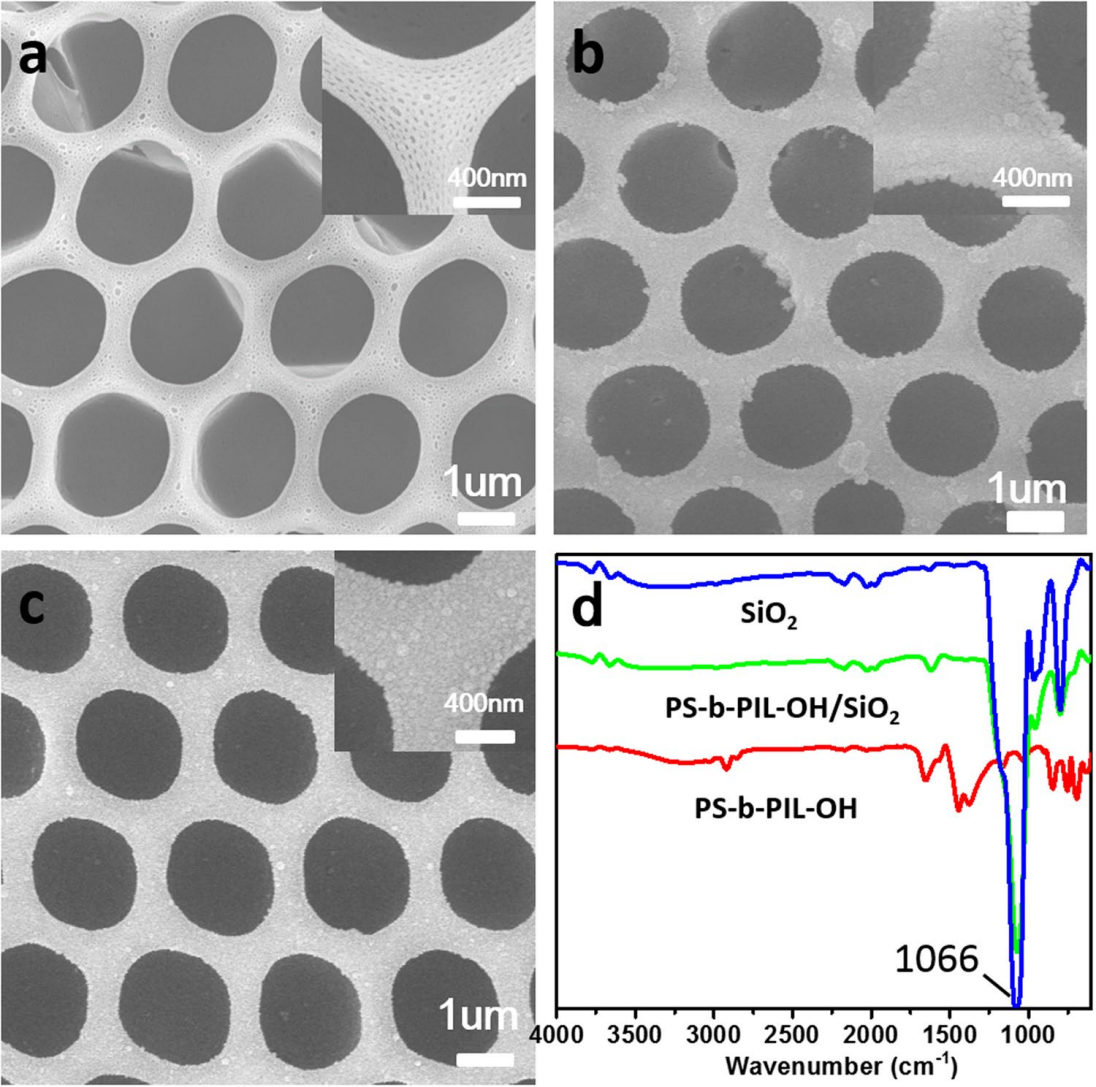
(**a**) SEM images of PS-b-PIL-OH honeycomb films from sample #4 PS190-b-PIL95. (**b**) SEM images of PS-b-PIL-OH/SiO_2_ composites. (**c**) SEM images of SiO_2_ honeycomb structures. (**d**) ATR-FTIR spectrum of PS-b-PIL-OH honeycomb films, PS-b-PIL-OH/SiO_2_ composite films and SiO_2_ honeycomb films.

PS-b-PIL-OH@SiO_2_ composite films were successfully fabricated in our work (Fig. 13b). Attributed to the ionic exchange by OH^−^, the films were grafted with base catalytic groups. Afterwards, we got the regular SiO_2_ honeycomb structured films. (Fig. 13c). The well-kept honeycomb structure proved that it is a very convenient and extendible approach to extend honeycomb films. ATR-FITR clearly confirms the successful hydrolysis of TEOS (Fig. 13d). For the PS-b-PIL-OH@SiO_2_ composite films and SiO_2_ honeycomb films, the characteristic peak of Si-O-Si asymmetric stretching vibration appeared at 1066 cm^−1^. And the characteristic peaks of -OH absorption peak of Si-OH appeared at 3000 cm^−1^ to 3600 cm^−1^. The characteristic peak of Si-O-Si symmetric stretching vibration and SiO_2_ tetrahedron also appeared at 960 cm^−1^ and 784 cm^−1^.

**Figure 14:**
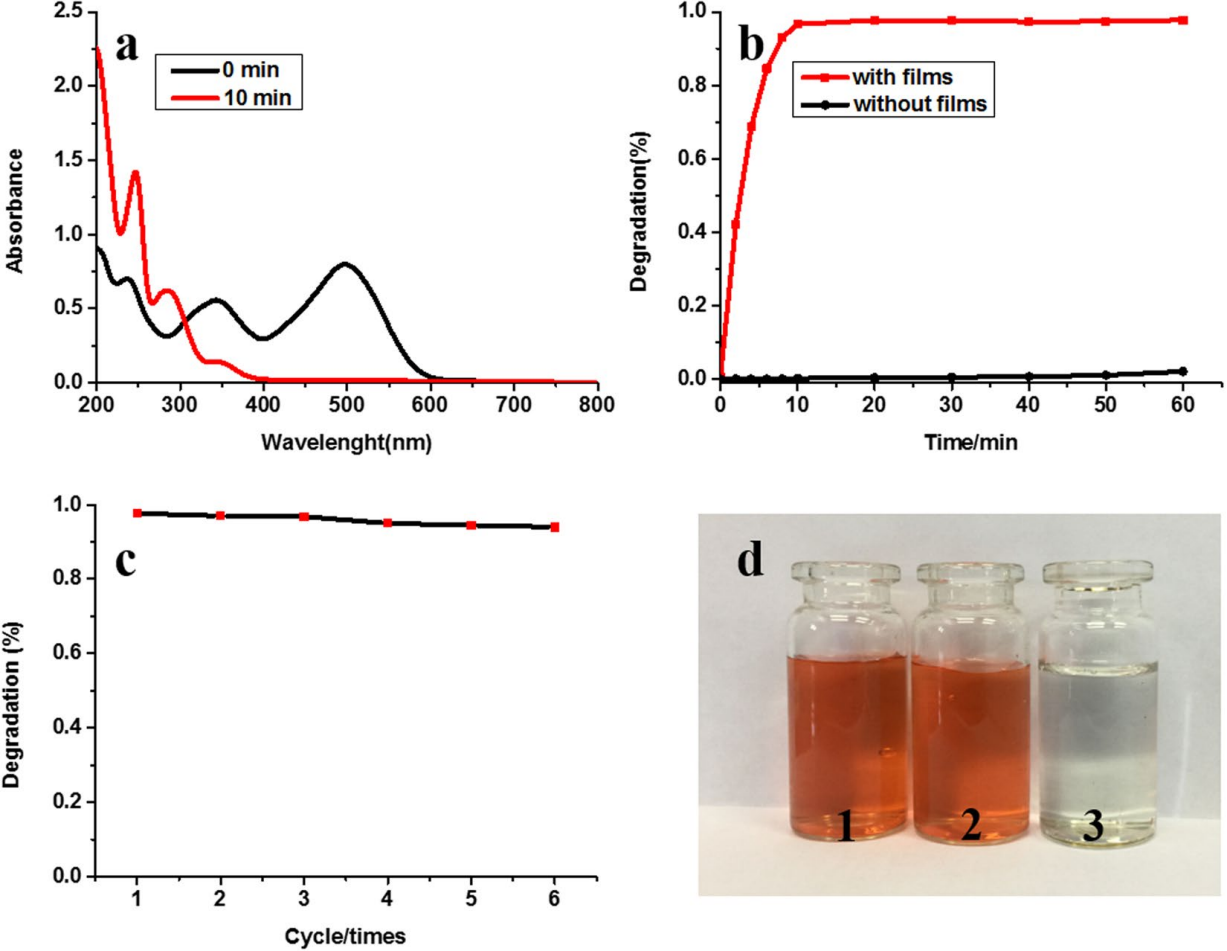
(**a**) UV-Vis spectra of the photocatalytic degradation of CR by PS-b-PIL-OH@PDA@Au. (**b**) Representative time-dependent degradation for photocatalytic reduction of CR by NaBH_4_ with and without gold films. (**c**) Degradation efficiency of membrane for six consecutive cycles. (**d**) Photographs of (1) original CR solution and CR solution after photocatalytic (2) in the absence of gold films (3) in the presence of gold films.

Due to common commercial use of Congo red as a colorant in dyeing industry, the research of its degradation is necessary^37,38^. Herein honeycomb films decorated with Au nanoparticles were used to photocatalytic degradation of Congo red dye. After 10 min, the concentration of Congo red significantly decreased, showing an obvious degradation (Fig. 14a). From Fig. 14b, the degradation of CR by our functional films fast reached maximum value with high efficiency while the degradation in the control experiment without adding films is almost zero. In order to research the stability of the films, we studied degradation efficiency of membrane for six consecutive cycles. The photocatalytic efficiency keeps well after six cycles (Fig. 14c). The photographs of original CR solution and CR solution after photocatalytic degradation in the absences or presences of gold films are shown in Fig. 14d, and the difference is obvious.

## Conclusions

In summary, a series of polystyrene-b-poly (ionic liquid) (PS-b-PIL) block copolymers were synthesized by us, and their formation behaviour of honeycomb films by BFs approach under different conditions were systematically studied for the first time. It was found that all the synthesized PS-b-PIL copolymers could form well-defined honeycomb porous films, but their morphologies are strongly dependent on the using experimental conditions, including block ratio, solvent concentration, relative humidity, water and salt solution pretreatment. Remarkably, due to the salt feature of ionic liquid moiety and the resultant osmotic pressure as well as the micro-phase separation of block copolymer, the described PS-b-PIL are prone to form unique hierarchical structure compared to the conventional polymers. Importantly, we found that the anion exchange could provide an effective means, by which the as-synthesized BFs porous films from PS-b-PIL copolymers could be further converted to conveniently and facilely access other films. As a demonstration, a series of ordered porous films with different surface (hydrophilic and hydrophobic) property, porous SiO_2_ film, porous polydopamine films with decorated Au or glutathione was successfully prepared by using different counteranions as well as further conversion. Due to the unlimited combination of cation and anion in ionic liquid moiety, all the results described here indicate that the BFs films generated by using PS-b-PIL could serve as a platform to access various functional porous films by a simple counteranion exchange. Especially, based on the concept of “task-specific” ionic exchange, we can introduce varieties of functional anions into PS-b-PIL BFs porous films by anion-exchange reactions, leading to a broad spectrum of new porous films with desired functions. Thus, we believe the method reported here would provide a new strategy with a great flexibility and expendability to access different BFs porous films, which cannot be directly generated by using BFs approach.

## Methods

### Materials and methods

Styrene, vinyl benzyl chloride, 2,2-azobisisobutyronitrile (AIBN), ethanol, glutathione (GSH), dopamine, chloroauric acid, sodium hydroxide, TEOS, Congo red, deionized water, chloroform and DMF were bought from the chemical suppliers. Unless otherwise stated, all the chemicals were used without further purification. Vinyl benzyl chloride and styrene were distilled under reduced pressure before use. AIBN was purified by recrystallization three times from ethanol. Glass substrates were cut to 1 × 1 cm^2^ and were cleaned prior to use in piranha solution (7:3 v/v of H_2_SO_4_/H_2_O_2_) and washed three times with deionized water and ethanol, then dried with N_2_.

### Synthetic procedures

#### Synthesis of PS-b-PIL block copolymer

The synthesis of a variety of poly (ionic liquid) block copolymers with imidazolium-functionalization was carried out according to reported procedures, denoted PS-b-PIL^19^. Firstly, polystyrene-b-poly (chloromethyl styrene) (PS-b-PCMS) block copolymers created by the stepwise RAFT of styrene and chloromethyl styrene (CMS)^20–23^. Secondly, PS-b-PCMS were quaternized here to get an imidazolium-functionalized charged block with a certain concentration of methylimidazole in DMF at 80 °C^24,25^. The characterization of M_n_ value and polydispersity for these samples was performed by ^1^H NMR and GPC.

#### Fabrication of hierarchically structured polymer films

The synthesized PS-b-PIL here was dissolved in mixed solvent (chloroform/methanol (20:1, v/v)), and magnetic stirred to get 0.3 wt% homogeneous solution. The solutions were filtered by 0.1 μm Millipore membrane before use. The preparation procedure of BFs process was carried out in a constant temperature and a built-in electric fan was used to adjust the humidity. About 30 μL solution was dropped on glass substrate under certain relative humidity conditions at 25 °C. After solvent evaporate completely, the regular porous honeycomb films were removed from the chamber. The films were allowed to air-dry in the oven overnight at room temperature. To the PS-b-PIL solution was added a certain amount of deionized water or sodium chloride saturated solution to yield 0.3 wt% polymer solutions with different water content and osmotic pressure after magnetic stirring for several hours. The following steps are the same as above.

#### Fabrication of hierarchically structured polymer films with different counter anions

Honeycomb PS-b-PIL films with different counter anions were obtained via anion exchange. Original films with chloride form were soaked in the corresponding salt aqueous solution such as potassium hydroxide solution (0.1 mol/L), sodium tetrafluoroborate solution (0.1 mol/L) and bistrifluoromethanesulfonimide lithium solution (0.1 mol/L) at ambient temperature for 24 h or 48 h. The converted films were washed with deionized water for three times. After washing, films were put into oven at 40 °C overnight and then films with OH^−^, BF_4_^−^ and Tf_2_N^−^ were gotten.

#### Fabrication of functional honeycomb structured films

PIL with OH^−^ honeycomb porous films were put into 0.2 mg/mL dopamine aqueous solution overnight. Then these films were washed with deionized for three several times. Honeycomb films coated with polymerized dopamine (PDA) were put into 0.02 mol/L chloroauric acid aqueous solution overnight. The pH value was adjusted to 8 with the use of triethylamine. The obtained films were washed several times with water, and then dried in the oven. Honeycomb films coated with PDA were put into 0.02 mol/L glutathione aqueous solution overnight. The pH value was also adjusted to 8 with the use of triethylamine. The obtained films were washed several times with water, and then dried in the oven.

#### Fabrication of ordered SiO_2_ honeycomb structures

The ordered SiO_2_ honeycomb structures were fabricated by a simple process. Tetraethyl orthosilicate (0.52 g) was dissolved in absolute ethyl alcohol (5 g), and then deionized water (1.08 g) was dripped into the above mixture solution. After mixing uniformly, honeycomb films of PS-b-PIL-OH were immersed in the above solution. After 48 h, the films were taken out and washed with absolute ethyl alcohol for three times. The SiO_2_/PS-b-PIL-OH composite films were immersed in CH_2_Cl_2_ solution to remove the PS-b-PIL. The obtained SiO_2_ honeycomb structures were washed with CH_2_Cl_2_ for three times, and then dried in oven.

#### Degradation of azo dyes

The photocatalytic application of the honeycomb films coated with gold nanoparticles was used for Congo red degradation. Films coated with gold nanoparticles (10 mg) and excess NaBH_4_ (110 mg) were put in 200 mL CR solution (100 mg/mL) in conical flask with magnetic stirring. After uniform mixing, put the above sample under ultraviolet light. The control experiments were performed in the group without the addition of the catalytic films. The degradation rate of CR was calculated by the absorbance at 498 nm (maximum characteristic absorption peak of Congo red).

### Characterization

^1^H NMR spectra were obtained using a JEOL JNM-ECA300 at 300 MHz with CDCl_3_. Gel permeation chromatography (GPC) was performed by a set of a Waters 515 HPLC pump. The surface pore structure and morphologies of the honeycomb films were detected by a field emission scanning electron microscopy (FE-SEM, 10 kV, SU-8010) with energy dispersive X-ray spectrometry (EDS, 15 kV). Film samples were pre-coated with platinum before observed. The elements on the films were determined by SEM-EDS. Membrane morphologies and structures were studied by transmission electronic microscope (TEM, 80 KV, JEM 2010). The chemical structures of the coated polymer films were characterized by attenuated total reflection-Fourier transform infrared spectra in the range of 600–4000 cm^−1^ (ATR-FTIR, Bruker VERTEX70). The films were pre-treated in oven at 80 °C all-night before use. UV-Vis spectra were observed on PerkinElmer Lambda35 spectrometer.

## References

[1] Widawski G, Rawiso M, François B (1994). Self-organized honeycomb morphology of star-polymer polystyrene films. Nature.

[2] Liu C, Gao C, Yan D (2007). Honeycomb-Patterned photoluminescent films fabricated by self-Assembly of hyperbranched polymers. Angew. Chem. Int. Ed..

[3] Wan LS, Li JW, Ke BB, Xu ZK (2012). Ordered microporous membranes templated by breath figures for size-selective separation. J. Am. Chem. Soc..

[4] Sun XC, Bruckner C, Nieh MP, Lei Y (2014). A fluorescent polymer film with self-assembled three-dimensionally ordered nanopores: preparation, characterization and its application for explosives detection. J. Mater. Chem. A.

[5] Maruyama N (1998). Mesoscopic patterns of molecular aggregates on solid substrates. Thin Solid Films.

[6] Stenzel MH, Davis TP, Fane AG (2003). Honeycomb structured porous films prepared from carbohydrate based polymers synthesized via the RAFT process. J. Mater. Chem..

[7] Ma HM, Hao JC (2011). Ordered patterns and structures via interfacial self-assembly: superlattices, honeycomb structures and coffee rings. Chem. Soc. Rev..

[8] Wang W (2017). Deterministic reshaping of breath figure arrays by directional photo manipulation. ACS Appl. Mater. Interfaces.

[9] Wrzecionko E (2017). Variations of polymer porous surface structures via the time-sequenced dosing of mixed solvents. ACS Appl. Mater. Interfaces.

[10] Bera S, Pal M, Sarkar S, Jana S (2017). Hierarchically structured macro with nested mesoporous zinc indium oxide conducting film. ACS Appl. Mater. Interfaces.

[11] Zhang AJ, Bai H, Li L (2015). Breath figure: a nature-inspired preparation method for ordered porous films. Chem. Rev..

[12] Erdogan B (2004). Permanent bubble arrays from a cross-linked poly (para-phenyleneethynylene): picoliter holes without microfabrication. J. Am. Chem. Soc..

[13] Gong JL (2012). Fabrication of multi-level carbon nanotube arrays with adjustable patterns. Nanoscale.

[14] Li L (2009). Honeycomb-patterned hybrid films and their template applications via a tunable amphiphilic block polymer/inorganic precursor system. Chem. Mater..

[15] Hirai YJ, Yabu HS, Matsuo Y, Ijiro K, Shimomura M (2010). Arrays of triangular shaped pincushions for SERS substrates prepared by using self-organization and vapor deposition. Chem. Commun..

[16] Qian WJ, Texter J, Yan F (2017). Frontiers in poly (ionic liquid)s: syntheses and applications. Chem. Soc. Rev..

[17] Yuan JY, Schlaad H, Giordano C, Antonietti M (2011). Double hydrophilic diblock copolymers containing a poly (ionic liquid) segment: controlled synthesis, solution property, and application as carbon precursor. Eur. Polym. J..

[18] Yuan JY, Antonietti M (2011). Poly (ionic liquid)s: polymers expanding classical property profiles. Polymer.

[19] Haven JJ (2014). One pot synthesis of higher order quasi-block copolymer libraries via sequential RAFT polymerization in an automated synthesizer. Polym. Chem..

[20] Stancik CM, Lavoie AR, Achurra PA, Waymouth RM, Gast AP (2004). A neutron scattering study of the structure and water partitioning of selectively deuterated copolymer micelles. Langmuir.

[21] Ye YS, Sharick S, Davis EM, Winey KI, Elabd YA (2013). High hydroxide conductivity in polymerized ionic liquid block copolymers. ACS Macro Lett..

[22] Weber RL (2011). Effect of nanoscale morphology on the conductivity of polymerized ionic liquid block copolymers. Macromolecules.

[23] Stancik CM (2004). Micelles of imidazolium-functionalized polystyrene diblock copolymers investigated with neutron and light scattering. Langmuir.

[24] Carrasco PM (2011). Influence of anion exchange in self-assembling of polymeric ionic liquid block copolymers. Macromolecules.

[25] Sudre G, Inceoglu S, Cotanda P, Balsara NP (2013). Influence of bound ion on the morphology and conductivity of anion-conducting block copolymers. Macromolecules.

[26] Wong KH, Davis TP, Barner-Kowollik C, Stenzel MH (2007). Honeycomb structured porous films from amphiphilic block copolymers prepared via RAFT polymerization. Polymer.

[27] Gong JL, Xu BG, Tao XM (2017). Breath figure micromolding approach for regulating the microstructures of polymeric films for triboelectric nanogenerators. ACS Appl. Mater. Interfaces.

[28] Bae J, Russell TP, Hayward RC (2014). Osmotically driven formation of double emulsions stabilized by amphiphilic block copolymers. Angew. Chem. Int. Ed..

[29] Guo JN, Zhou YX, Qiu LH, Yuan C, Yan F (2013). Self-Assembly of amphiphilic random co-poly(ionic liquid)s: the effect of anions, molecular weight, and molecular weight distribution. Polym. Chem..

[30] Lin BC, Qiu LH, Qiu B, Peng Y, Yan F (2011). A soluble and conductive polyfluorene ionomer with pendant imidazolium groups for alkaline fuel cell applications. Macromolecules.

[31] Si ZH, Sun Z, Gu FL, Qiu LH, Yan F (2014). Alkaline stable imidazolium-based ionomers containing poly (arylene ether sulfone) side chains for alkaline anion exchange membranes. J. Mater. Chem. A.

[32] Qiu B, Lin BC, Qiu LH, Yan F (2012). Alkaline imidazolium- and quaternary ammonium-functionalized anion exchange membranes for alkaline fuel cell applications. J. Mater. Chem..

[33] Raj SG, Kumara GR, Mohana R, Pandia S, Jayavel R (2005). Structural, optical and dielectric studies on solution-grown semi-organic L-kistidine tetrafluoroborate single crystals. Mater. Chem. Phys..

[34] Li MT (2013). New polymerized ionic liquid (PIL) gel electrolyte membranes based on tetraalkylammonium cations for lithium ion batteries. J. Membrane Sci..

[35] Salgado R, Rio RD, Valle MA, Armijo F (2013). Selective electrochemical determination of dopamine, using a poly (3, 4-ethylenedioxythiophene)/polydopamine hybrid film modified electrode. J. Electroanal. Chem..

[36] Polshettiwar V, Varma RS (2010). Nano-Organocatalyst: magnetically retrievable ferrite-anchored glutathione for microwave-assisted Paal-Knorr reaction, Aza-Michael addition, and pyrazole synthesis. Tetrahedron.

[37] Sahoo A, Tripathy SK, Dehury N, Patra S (2015). A porous trimetallic Au@Pd@Ru nanoparticle system: synthesis, characterisation and efficient dye degradation and removal. J. Mater. Chem. A.

[38] Tripathi BP, Dubey NC, Choudhury S, Formanek P, Stamm M (2015). Ultrathin and switchable nanoporous catalytic membranes of polystyrene-b-poly-4-vinyl pyridine block copolymer spherical micelles. Adv. Mater. Interfaces.

